# Adjunctive Intravenous Dexmedetomidine’s Effect on Surgical Visualization and Analgesia in Arthroscopic Bankart Repair: A Retrospective Cohort Study

**DOI:** 10.3390/jcm15155907

**Published:** 2026-07-29

**Authors:** Resul Bircan, Semih Yaş, Selin Bağcaz, İrfan Güngör, Gökçen Emmez, Ulunay Kanatlı

**Affiliations:** 1 Orthopedics and Traumatology Department, Ankara Oncology Training and Research Hospital, 06560 Ankara, Türkiye; drsemihyas@gmail.com; 2 Anesthesiology & Reanimation Department, Mamak State Hospital, 06270 Ankara, Türkiye; selinsamsun@hotmail.com; 3 Anesthesiology & Reanimation Department, Gazi University Medical Faculty Hospital, 06560 Ankara, Türkiye; irfan.gungor@gazi.edu.tr (İ.G.); gokcenemmez@gazi.edu.tr (G.E.); 4 Orthopedics and Traumatology Department, Gazi University Medical Faculty Hospital, 06560 Ankara, Türkiye; ukanatli@gazi.edu.tr

**Keywords:** dexmedetomidine, Bankart, postoperative pain, shoulder dislocation

## Abstract

**Background/Objectives**: Optimizing surgical visualization via controlled hypotension and managing postoperative pain remain challenging in arthroscopic Bankart repair. While interscalene brachial plexus block (ISBPB) is effective, pharmacological adjuncts may further improve hemodynamics and prolong analgesia. The aim of this study was to assess the impact of intraoperative intravenous dexmedetomidine on visual clarity, hemodynamics, and postoperative pain during arthroscopic Bankart repair. **Methods**: This retrospective, single-center cohort study evaluated 63 patients undergoing isolated anterior Bankart repair under ISBPB. The dexmedetomidine group (Group D, *n* = 33) received an intravenous dexmedetomidine loading dose (1 mcg/kg) and maintenance infusion (0.5 mcg/kg/h) alongside ISBPB. A historical control group (Group C, *n* = 30) received ISBPB alone. Secondary outcomes included serial intraoperative hemodynamics, blinded surgeon-rated surgical visualization, and pain scores, while the primary outcome was postoperative analgesia duration. **Results**: Baseline characteristics were comparable between groups. Group D yielded significantly higher surgeon satisfaction for visual clarity (*p* < 0.001). Intraoperatively, Group D showed a significantly lower diastolic blood pressure at the 50 min mark after correction for multiple comparisons. Postoperatively, Group D exhibited significantly lower 24 h visual analog scale (VAS) pain scores (*p* < 0.001) and a significantly prolonged duration of analgesia (7.45 vs. 4.16 h; *p* = 0.001). Overall patient satisfaction and Pain Catastrophizing Scale scores did not differ significantly between cohorts. **Conclusions**: In this retrospective cohort, adjunctive intravenous dexmedetomidine added to ISBPB was associated with improved surgeon-rated visualization, lower postoperative pain scores, and prolonged analgesia, without an increase in adverse events. Due to the retrospective design and historical control group, these findings should be interpreted as associations; prospective randomized trials are required to confirm them.

## 1. Introduction

Traumatic anterior glenohumeral instability is a prevalent and functionally limiting condition, with a particularly high incidence among young, physically active individuals. Participation in contact sports, activities involving repetitive overhead motion and male sex are significant risk factors for initial dislocation events [1]. The principal pathological consequence of an anterior dislocation is the avulsion of the anteroinferior glenoid labrum and the attached inferior glenohumeral ligament complex from the glenoid rim, a lesion first described by Bankart in 1923 [2]. This disruption of the primary static stabilizers of the shoulder joint predisposes the patient to subsequent episodes of instability [3].

While non-operative management may be appropriate for some patients, the rate of recurrent instability is prohibitively high, especially in younger cohorts, with more than 80% of patients under the age of 20 experiencing a subsequent dislocation [4]. Consequently, surgical intervention is frequently indicated for patients under the age of 25 and patients with recurrent symptomatic instability or for those who wish to return to high-demand activities [5]. Over the past two decades, arthroscopic Bankart repair has evolved to become the gold standard for the surgical management of anterior shoulder instability in patients without critical glenoid bone loss (generally defined as less than 12–25%) or a Hill–Sachs lesion [4,5].

One of the main challenges of arthroscopic Bankart surgery is the loss of visual clarity caused by bleeding during anterior labrum release or the debridement of fibrotic tissues covering the glenoid edge [6,7]. This problem can prolong operative time and potentially compromise implant placement [8]. Another challenge is effective perioperative and postoperative pain management, which is a critical component of recovery after arthroscopic instability surgery. The recent literature demonstrates a shift toward multimodal analgesia strategies, which combine non-opioid analgesics and regional anesthesia to achieve superior pain relief with reduced opioid consumption [9,10]. Within this paradigm, regional anesthesia, particularly the interscalene brachial plexus block (ISBPB), has become a cornerstone, providing profound site-specific analgesia [11].

Dexmedetomidine, a highly selective alpha-2 adrenergic agonist, has emerged as a potent adjuvant in this setting. Pharmacologically, it acts centrally on the locus coeruleus to produce sedation and anxiolysis, and on the spinal cord to modulate nociceptive transmission, providing analgesia without significant respiratory depression [12,13]. Furthermore, its sympatholytic properties reduce sympathetic outflow, leading to a dose-dependent reduction in heart rate and blood pressure, which is theoretically advantageous for controlled hypotension in arthroscopic procedures.

While the efficacy of perineural dexmedetomidine as an additive to nerve blocks is well-documented, the utility of systemic intravenous (IV) dexmedetomidine in conjunction with standard regional anesthesia remains an area of active investigation [14,15]. Recent evidence suggests that IV administration may provide comparable analgesic benefits to perineural administration while offering superior hemodynamic stability [16]. This study aimed to evaluate the efficacy of intraoperative intravenous dexmedetomidine infusion on intraoperative hemodynamics, postoperative pain, and surgeon satisfaction in patients undergoing arthroscopic Bankart repair. We hypothesized that systemic dexmedetomidine synergizes with the ISBPB to prolong analgesia and provide stable controlled hypotension, thereby improving surgical conditions.

## 2. Materials and Methods

This was a retrospective, single-center cohort study. The study protocol was approved by the Gazi University Faculty of Medicine Clinical Research Ethics Committee (date: 5 March 2026, no: 2026-349). The study was conducted in accordance with the principles of the Declaration of Helsinki. This single-center, retrospective study was conducted at the Medicine Faculty of Gazi University, Department of Orthopedics and Traumatology between 2022 and 2025. All clinical data analyzed in this study—including hemodynamic measurements, visual analog scale (VAS) pain scores, Pain Catastrophizing Scale (PCS) scores, duration of analgesia, and archived arthroscopic video recordings—were generated as part of routine clinical care during the study period. Consecutive eligible patients operated on between March 2022 and June 2025 were screened for inclusion. The study period began in 2022 because standardized, routine recording of intraoperative hemodynamic parameters, VAS pain scores, PCS scores, and duration of analgesia was implemented as departmental practice at that time, ensuring uniform and complete data capture for all included patients.

### 2.1. Study Design and Population

Inclusion criteria were all patients with ASA physical status I–II and skeletal maturity, comprising adolescents and adults aged 15 years or older who underwent arthroscopic repair of an isolated anterior Bankart lesion. Exclusion criteria included history of hypersensitivity to dexmedetomidine, coagulopathy, cardiac anomalies, hypertension requiring medication or chronic opioid use, use of additional intraoperative analgesic drugs, receipt of a dexmedetomidine dose other than the standardized regimen, and incomplete hemodynamic or outcome records. All patients undergoing arthroscopic shoulder surgery in our clinic routinely receive an ISBPB using 25 mL of 0.5% bupivacaine together with 0.05 mg/kg intravenous midazolam for sedation before the block. Since 2023, intravenous dexmedetomidine has been part of routine institutional practice for these procedures: unless contraindicated or unavailable due to supply constraints, patients received an intravenous dexmedetomidine loading dose of 1 mcg/kg administered over 10 min, followed by a maintenance infusion of 0.5 mcg/kg/h until the end of surgery. Group allocation in this study was therefore determined solely by the calendar period of surgery rather than by patient- or surgeon-level decisions: patients operated from 2023 onward, when dexmedetomidine had become routine, formed the dexmedetomidine group (Group D), whereas patients operated before this date, when supplementary dexmedetomidine was not part of routine practice, formed the control group (Group C). Patients from the dexmedetomidine era who did not receive the drug purely because of a documented contraindication or a temporary supply shortage were not reassigned to the control group but were excluded, so that allocation would not be confounded by clinical contraindications.

**Figure 1:**
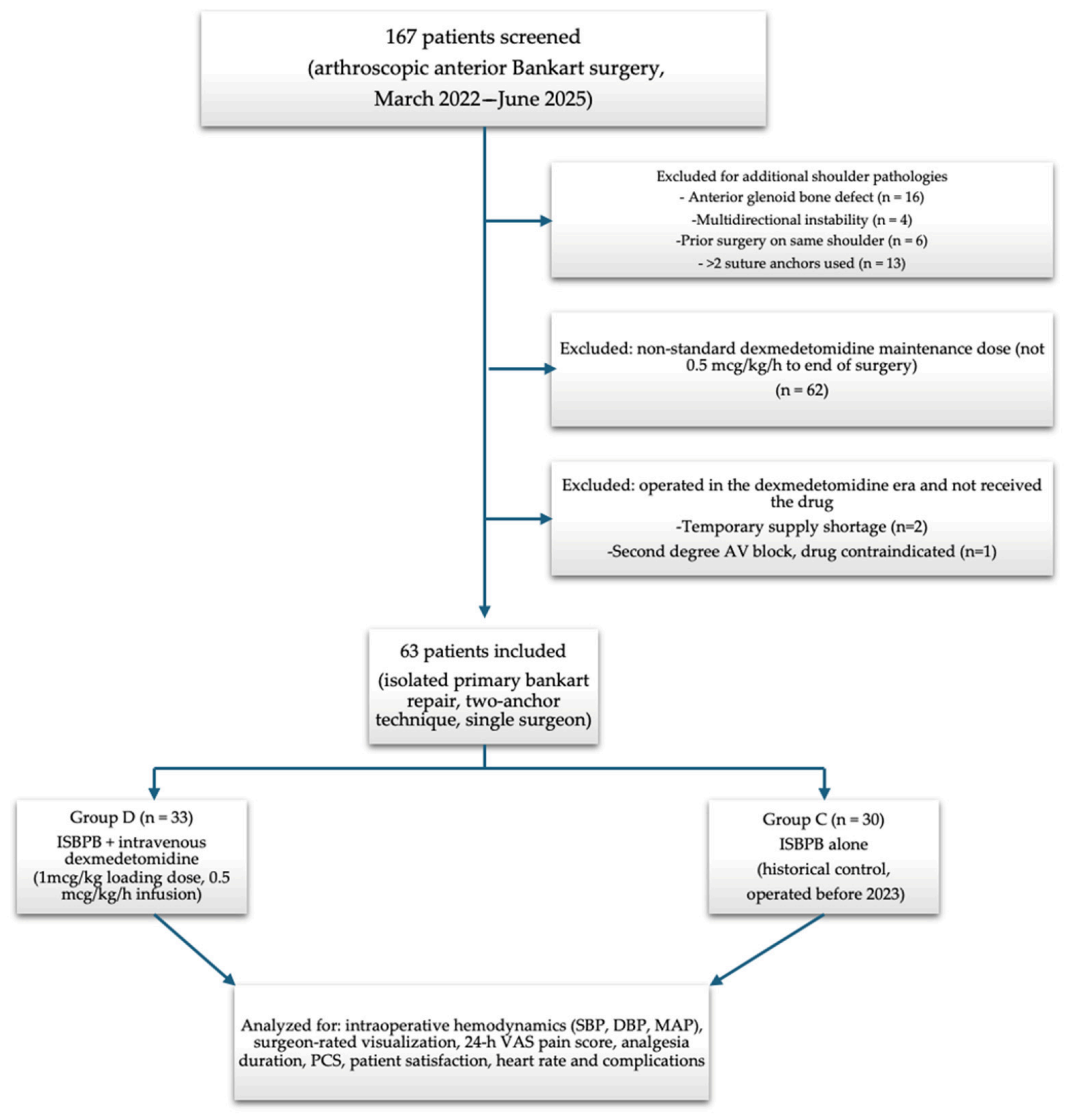
STROBE-style flow diagram of patient selection. ISBPB, interscalene brachial plexus block.

A total of 167 patients who had undergone arthroscopic anterior Bankart surgery during the study period were screened. Patients with other shoulder pathologies (SLAP lesion, rotator cuff tear, etc.) that require intervention had already been excluded before this screening step. Three patients operated on during the dexmedetomidine era did not receive the drug and were excluded: two because of a temporary supply shortage, and one with a history of second-degree atrioventricular block, in whom dexmedetomidine was contraindicated. During eligibility assessment, 16 patients with an anterior glenoid bone defect, four with multidirectional instability, six with prior surgery on the same shoulder, and 13 in whom more than two suture anchors were used for the repair were excluded, to retain a homogeneous population of isolated primary Bankart repairs performed with a standardized two-anchor technique. A further 62 patients from the dexmedetomidine era were excluded because they did not receive the standardized maintenance regimen: although they received dexmedetomidine, their maintenance infusion was titrated to a higher or lower rate than the protocol dose of 0.5 mcg/kg/h continued until the end of surgery, most commonly to adjust the depth of sedation according to body habitus and baseline heart rate. Restricting the analysis to patients who received the identical standardized regimen ensured a pharmacologically homogeneous dexmedetomidine group. No patient was excluded because of missing hemodynamic or outcome data. After these steps, 63 patients with no relevant comorbidity or medication history and an isolated anterior Bankart lesion remained, comprising 33 patients in Group D (ISBPB plus intravenous dexmedetomidine) and 30 patients in Group C (ISBPB alone). The patient selection process is summarized in the STROBE-style flow diagram (Figure 1). All operations were performed by a single senior shoulder surgeon using two suture anchors for the repair, with the patient in the lateral decubitus position and a constant arthroscopic pump pressure of 60 mmHg.

### 2.2. Data Collection and Definitions

Data regarding patient history obtained from anesthesia assessment forms, ASA classifications, intraoperative anesthesia methods, and intraoperative hemodynamic parameters (systolic blood pressure, diastolic blood pressure (SBP and DBP), postoperative heart rate (recorded in the recovery room) and postoperative complications (Horner syndrome, respiratory problems, hoarseness) were recorded. If the surgery concluded before the 60 min mark, blood pressure measurements from subsequent time points were excluded from the analysis, as patient movement off the operating table could confound the readings. Comparisons were made between patients receiving intraoperative intravenous dexmedetomidine infusion and those who did not, regarding intraoperative hemodynamic data at 0 (baseline), 10, 20, 30, 40, 50 and 60 min. Data collected as part of the routine clinical protocol, such as postoperative 24th hour VAS and PCS scores, duration of analgesia, defined as the time elapsed (in hours) from performance of the block to the patient’s first request for rescue analgesics (1 g intravenous paracetamol), as well as postoperative 24th hour patient satisfaction, were also compared. Throughout the study period, postoperative analgesia followed a single standing institutional protocol that did not change between the two study periods: postoperative analgesia consisted of intravenous paracetamol 1 g administered on a patient-request basis, with a minimum interval of 6 h between doses, according to fixed nursing criteria (a reported pain score above a predefined threshold) rather than at scheduled intervals or at nursing discretion. Accordingly, the duration of analgesia was defined as the time from performance of the block to the patient’s first request for intravenous paracetamol. The time of the first rescue request was documented prospectively in the nursing records as part of routine care. Because the same ward, nursing team, and written protocol applied to both groups, differences in nursing practice or institutional protocol are unlikely to account for the observed between-group difference, although individual variation in pain tolerance and expectation cannot be excluded and is acknowledged as a limitation.

**Figure 2:**
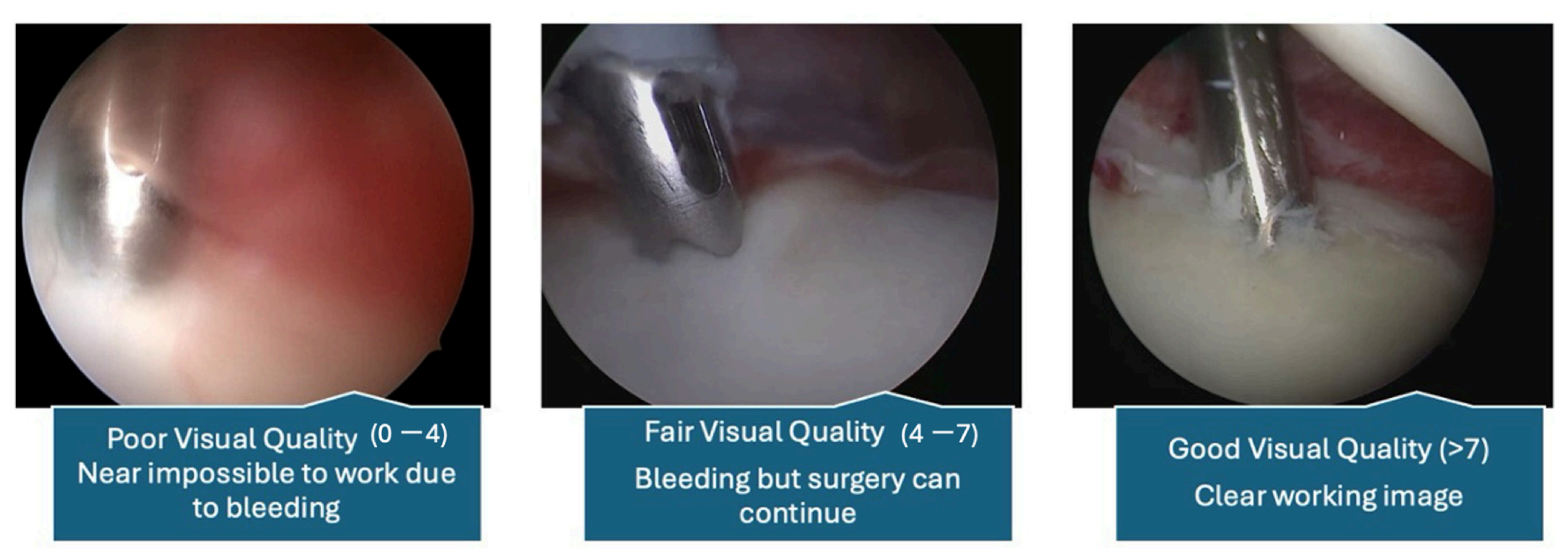
Arthroscopic clarity of visual field classification system used with examples.

Visual clarity during the labral-release phase of surgery was assessed retrospectively from the archived arthroscopic video recordings by two shoulder surgeons (T.K.E. & T.A.), each with at least 5 years of shoulder arthroscopy experience. Both assessors were blinded to group allocation, and a 0–10 visual analog scale (VAS) was used for scoring, adapted from the visualization grading used by van Montfoort et al. in a randomized trial of irrigation-fluid clarity in shoulder arthroscopy [17]. A field in which bleeding made arthroscopic work almost impossible was rated poor (0–4), a field in which bleeding was present, but surgery could continue was rated fair (4–7), and a consistently clear working image was rated good (>7) (Figure 2). Interobserver and intraobserver reliability (Cohen’s kappa, κ, with 95% confidence intervals) were calculated from these independent, pre-consensus scores, so that the reported agreement reflects genuine assessor concordance rather than the post-consensus classification. Only after reliability had been quantified were any remaining discrepancies resolved in a consensus meeting; the resulting consensus classification was then used for the between-group comparison of visualization category. Agreement strength was interpreted using conventional criteria (κ = 0.81–1.00 considered almost perfect agreement), and statistical significance was set at *p* < 0.05.

Mean arterial pressure (MAP) was later calculated using the “[(2×DBP)+SBP]”÷3 formula. PCS is an instrument designed to assess the thoughts and feelings individuals experience in response to pain. It consists of 13 items rated on a 5-point Likert-type scale (0 = not at all, 4 = all the time), with a total score ranging from 0 to 52. The Turkish version of the PCS, validated by Ugurlu et al., was used in this study [18]. Patient satisfaction was assessed again using a VAS. Surgeon satisfaction was rated immediately after surgery with regard to the quality of arthroscopic visualization on a scale of 1–10.

Generative artificial intelligence tools were utilized during the preparation of this manuscript. Specifically, Gemini 3.5 was used to shorten the abstract to meet the journal’s word count requirements, and ChatGPT 5.5 was utilized for recommendations regarding the preparation of the graphical abstract.

### 2.3. Statistical Analysis

All analyses were performed using IBM SPSS Statistics, version 21.0. The duration of postoperative analgesia was pre-specified as the primary outcome; surgeon-rated visualization, 24 h VAS pain score, intraoperative hemodynamic parameters (SBP, DBP, and MAP), postoperative heart rate, PCS score, and patient satisfaction were treated as secondary outcomes. Descriptive statistics were presented as frequencies, percentages, means with standard deviations, and medians with interquartile ranges. Normality was tested with the Kolmogorov–Smirnov test and Shapiro–Wilk test. Continuous variables that were normally distributed (age, weight, 24 h VAS pain score, duration of analgesia, PCS score, postoperative heart rate, and SBP, DBP, and MAP values) were compared using the independent-samples *t*-test, whereas variables that departed from normality (surgeon satisfaction, patient satisfaction, and the individual time points flagged in the tables) were compared using the Mann–Whitney U test; the test applied to each variable is indicated in the corresponding table footnote. For categorical variables, the Chi-square test was used. To reduce the risk of type I error arising from repeated testing of the intraoperative hemodynamic parameters at seven time points, the significance threshold for each hemodynamic family (SBP, DBP, and MAP) was additionally interpreted against a Bonferroni-corrected level of 0.05/7 ≈ 0.007, and both the uncorrected and corrected interpretations are reported. For the pre-specified primary outcome and the principal secondary outcomes, 95% confidence intervals for the between-group difference are reported alongside *p*-values, so that interpretation does not rely on statistical significance alone. A two-sided *p*-value < 0.05 was considered statistically significant. The effect size of the interventions was calculated using Cohen’s d. A *d* value of less than 0.2 indicates a weak effect size, a value between 0.5 and 0.8 indicates a moderate effect size, and a value greater than 0.8 indicates a strong effect size.

For comparisons of two independent groups using the non-parametric Mann–Whitney U test, the effect size was calculated using the formula r=Z∕√N. Accordingly, r value of less than 0.3 indicates a small effect size, 0.3 to 0.5 indicates a moderate effect size, and greater than 0.5 indicates a strong effect size [19]. The negative effect size coefficients obtained from the analyses represent the mathematical direction of the effect; therefore, the absolute values of the coefficients were used to interpret the magnitude of the effect.

The study utilized a purposive sample comprising all consecutive, eligible patients who underwent isolated arthroscopic anterior Bankart repair under ISBPB at our institution between March 2022 and June 2025. Because the cohort was fixed and defined by these dates, no a priori sample-size calculation determined the number of patients available for analysis; the precision of each estimate is instead conveyed by the 95% confidence intervals reported for the primary and principal secondary outcomes.

## 3. Results

When age, weight, and ASA scores were analyzed, no statistically significant differences were found between the two groups (Group D and C) (*p* = 0.965, *p* = 0.711, and *p* = 0.211, respectively) (Table 1).

Surgeon satisfaction, postoperative VAS scores, and duration of analgesia were significantly superior in the patients who received dexmedetomidine (*p* < 0.001, *p* < 0.001, and *p* = 0.001, respectively) (Table 1). With an r value of −0.518 indicating a strong effect, surgeon-rated satisfaction was higher in the dexmedetomidine group than in the control group (mean difference 1.19 points; 95% CI 0.66 to 1.72). For the 24 h VAS pain score, patients in the dexmedetomidine group reported lower pain (mean difference −2.26 points; 95% CI −2.87 to −1.65; Cohen’s d = 1.88), and the duration of analgesia was longer (mean difference 3.29 h; 95% CI 1.35 to 5.23; Cohen’s d = 0.85), both representing large effect sizes. However, no significant differences were observed between the two groups regarding patient satisfaction and PCS scores (*p* = 0.383 and *p* = 0.810, respectively) (Table 1). Heart rate measurement recorded at the end of the operation was statistically significantly lower in the group that had dexmedetomidine with a strong effect (69.4 vs. 80.2 bpm; mean difference −10.74 bpm, 95% CI −15.69 to −5.79; *p* < 0.001; Cohen’s d = 1.08).

For visual clarity classification, interobserver agreement was excellent (κ = 0.807, 95% CI 0.725–0.884), while intraobserver agreement was substantial to excellent (κ = 0.778, 95% CI 0.687–0.856). Overall, visual clarity classification demonstrated high reproducibility, supporting the consistency of the assessment methodology used in this study. No patients experienced respiratory distress or hoarseness during the surgical procedure or within the first 24 h postoperatively. Horner’s syndrome was observed in only one patient who received an isolated ISBPB.

Table 2 and Table 3 show SBP and DBP recorded at 10 min intervals until the end of the first hour of surgery. While no significant differences were observed for SBP at any time point, uncorrected DBP values demonstrated significant differences between the two groups from the 20 to the 50 min marks. When the Bonferroni-corrected threshold for seven comparisons (*p* ≈ 0.007) was applied, only the 50 min difference remained statistically significant (*p* = 0.003; mean difference −7.43 mmHg, 95% CI −12.18 to −2.69), whereas the 20-, 30-, and 40 min differences (*p* = 0.042, 0.015, and 0.036) did not survive correction and are therefore reported as exploratory. The uncorrected effect sizes for the 20-, 30-, and 40 min marks were moderate (*d* = 0.521, *d* = 0.637, and *d* = 0.561 respectively), while for the 50th minute the effect size was strong (*d* = 0.936).

Table 4 shows calculated MAP values of both groups. At the uncorrected level, there was a statistically significant difference at the 20-, 30-, and 50 min marks (*p* = 0.037, 0.032, and 0.008, respectively). After Bonferroni correction (*p* ≈ 0.007), none of these MAP differences reached statistical significance, so the MAP findings are presented as exploratory and hypothesis-generating; the corresponding between-group mean differences and confidence intervals were −4.26 mmHg (95% CI −8.30 to −0.22) at 20 min, −5.25 mmHg (95% CI −10.03 to −0.47) at 30 min, and −5.20 mmHg (95% CI −8.99 to −1.42) at 50 min. The uncorrected effect size was small for the 30th minute (r=0.271), moderate for the 20th minute (*d* = 0.535) and strong for the 50th minute (*d* = 0.822).

When surgeon satisfaction was categorized as poor, fair, and good, there was a statistically significant difference between the two groups, as seen in Table 5 (*p* = 0.003 and Fisher’s exact 0.005) (Table 5).

## 4. Discussion

In this retrospective cohort, adjunctive intravenous dexmedetomidine infusion was associated with a lower intraoperative diastolic blood pressure that remained significant at 50 min, more favorable surgeon-rated surgical conditions, and prolonged postoperative analgesia in patients undergoing arthroscopic Bankart repair under interscalene brachial plexus block. These observations are consistent with the hypothesis that systemic dexmedetomidine acts as a useful adjunct to regional anesthesia in arthroscopic Bankart surgery, plausibly through its sympatholytic effects, which may contribute both to optimization of the surgical field and to enhancement of postoperative analgesia. Because the design was observational and used a historical control group, however, these findings should be interpreted as associations rather than as evidence of a causal effect.

Surgeons rated the visual field as significantly clearer in the dexmedetomidine group (mean satisfaction ~9.4 vs. 8.2), consistent with the better controlled hypotension. These results closely align with previous reports. In 2017, Abdel-Hamid showed that IV dexmedetomidine (vs. fentanyl) markedly reduced mean arterial pressure and heart rate during shoulder arthroscopy, improving surgical-field visibility and surgeon satisfaction [20]. Similarly, Menshawi et al. (2020) found that dexmedetomidine achieved satisfactory controlled hypotension (equivalent to remifentanil) with prolonged postoperative analgesia and lower VAS [21]. Visual clarity can affect surgical time and the quality of the surgery. While experienced surgeons may manage this situation, for an inexperienced one, loss of visual quality can prolong the surgical time and lower the quality of the procedure.

In our series, the dexmedetomidine group maintained equivalent systolic blood pressure (no significant differences at any time point) while showing moderately lower DBP and MAP in mid- to late operative time points (20–50 min) at the uncorrected level, of which only the 50 min DBP difference survived correction for multiple comparisons—reflecting the alpha-2 sympatholytic effect that blunts vascular tone. Dexmedetomidine exhibits a biphasic hemodynamic response; at higher concentrations (such as during the bolus), peripheral alpha-2b receptors may cause vasoconstriction, maintaining systolic pressure, while central alpha-2a effects drive sympatholysis and bradycardia [12]. This hemodynamic profile appears advantageous for arthroscopic Bankart surgery, as it maintains perfusion pressure (SBP) while reducing the mean pressure and rate that drive capillary oozing.

The hemodynamic differences we observed were modest, on the order of 4 to 7 mmHg for DBP and MAP, and most did not survive correction for multiple testing. A difference in this magnitude is unlikely to act independently as a hemodynamic mechanism altering clinical visibility, so a physiological reading is more useful than a purely statistical one. In a closed, irrigated joint, the field is spoiled mainly by low-pressure capillo-venous oozing, which depends on the gradient between the irrigation pressure and the surrounding capillary and venous pressure. With the pump held at 60 mmHg in both groups, a lower diastolic and mean pressure narrows that gradient and reduces oozing, even when systolic pressure is unchanged. Dexmedetomidine is well suited for this purpose: it lowers blood pressure chiefly through central sympatholysis, reducing sympathetic outflow and circulating catecholamines rather than by directly dilating the vascular bed, and it additionally exerts a peripheral α2-mediated vasoconstrictive effect [12,22]. This distinguishes it from direct vasodilators such as nitroglycerin or sodium nitroprusside, which lower pressure but can paradoxically worsen surgical-field bleeding through vasodilation [23]. The lower heart rate in the dexmedetomidine group (69.4 vs. 80.2 bpm) is best read as a sign of this reduced sympathetic tone and pulsatility rather than an independent cause of hemostasis, as we did not measure cardiac output. This pattern—lower DBP, MAP, and heart rate alongside a better field—matches what has been reported with intravenous dexmedetomidine in endoscopic sinus surgery [23]. One caveat is specific to the shoulder: earlier work links visual clarity primarily to systolic pressure and to the gradient between systolic and intra-articular pressure, and we found no systolic difference between groups [24,25]. Comparable systolic pressures and identical pump settings therefore argue against a purely hemodynamic explanation and point instead to a combined effect of lower diastolic and mean pressures, reduced pulsatility, and the sympatholytic, anxiolytic state produced by dexmedetomidine. Because we did not measure bleeding volume or operative time, we regard the clinical relevance of these changes as biologically plausible rather than proven, pending confirmation with objective measures of bleeding.

Patients receiving dexmedetomidine (Group D) had significantly lower postoperative heart rates and extended analgesia duration (mean ~7.5 vs. 4.2 h) and much lower 24 h VAS pain scores than controls. There are multiple studies supporting these findings. In the meta-analysis by Lu et al. (2025), patients given dexmedetomidine with their block as IV or added to shoulder block had significantly longer analgesia and lower 24 h opioid use [16]. In the meta-analysis, the effect on blood pressure was reported as neutral, with a side note stating that the study by Abdallah et al. is causing heterogeneity for this result, and when excluded, there is still a significant increase in hypotension when dexmedetomidine is used [26]. In the 2018 study by Li et al., they showed that adding IV dexmedetomidine to ISBPB gives similar results in prolonging the analgesia time and lowering the heart rate [27]. In contrast to our findings, SBP was also affected in their study, which may be attributed to a higher dexmedetomidine maintenance dose (0.8 mcg/kg/h). In 2014, Bengisun et al. showed that using dexmedetomidine as a perineural adjunct for interscalene block in arthroscopic shoulder surgery also gives better postoperative VAS scores and longer analgesia duration [28]. Likewise, we saw a large effect on postoperative pain (VAS) and duration, but no increase in adverse events—only one transient Horner’s case in the control group. Thus, our findings are consistent with a beneficial adjunctive role for IV dexmedetomidine, although the observational design precludes causal inference.

Notably, some prior trials have reported that dexmedetomidine is comparable but not superior to interscalene block. Kanakalakshmi et al. (2022) randomized arthroscopic shoulder patients to IV dexmedetomidine infusion versus ultrasound-guided interscalene block [29]. They found equivalent intraoperative hemodynamic stability in both groups, but significantly better postoperative analgesia with the block (VAS lower, less opioid). In that study, dexmedetomidine was used instead of a block, so unsurprisingly block analgesia was superior. Our findings differ because dexmedetomidine was evaluated as an adjunct; indeed, the control group (block alone) experienced inferior pain relief and satisfaction compared to the combination group, demonstrating that systemic dexmedetomidine successfully complemented the regional block. This is consistent with other evidence that systemic dexmedetomidine can enhance the duration of bupivacaine ISBPB. For example, Rodrigues et al. (2021) reported that IV dexmedetomidine (50 μg) significantly prolongs block analgesia relative to control, though IV dexamethasone was even more potent [30]. In their trial, dexmedetomidine alone extended block duration by about 46% versus 59% for dexamethasone. Thus, our prolonged analgesia is in line with the notion that dexmedetomidine has a moderate adjunctive effect.

Dexmedetomidine’s use must balance benefits with risks. We found no increase in respiratory depression or major cardiac events. The classic side effects—bradycardia and hypotension—did not manifest dangerously at our moderate dose (1 μg/kg bolus, 0.5 μg/kg/h), but they have been noted in other studies. For instance, Albrecht et al. (2023) administered 1 μg/kg IV dexmedetomidine after dexamethasone + ISBPB and found significantly more intraoperative hypotension and earlier opioid requirement (shorter time to first morphine) compared to saline [31]. Similarly, Vanden Eede et al. (2025) compared escalating IV dexmedetomidine doses (1.0–2.0 μg/kg) after dexamethasone/ISBPB and saw no further prolongation of analgesia, but significant increases in bradycardia/hypotension and delayed extubation [32]. These contrasting findings suggest a ceiling effect: once dexamethasone and block are in place, extra dexmedetomidine may not add analgesia but will compound hemodynamic depression. In our cohort, the dose regimen (drawn from the anesthesia literature recommending 0.5–1.0 μg/kg loading, then 0.2–0.7 μg/kg·h) seemed optimal—maximizing the analgesic and sedation benefits without causing problematic bradycardia [14,32,33].

There were no differences in patient satisfaction or catastrophizing scores, which indicates that while dexmedetomidine improved pain and field quality, overall patient comfort was good in both groups (likely due to effective block). This is consistent with Kanakalakshmi et al. (2022), who reported comparable patient satisfaction between dexmedetomidine infusion and block [29].

This study relied entirely on records collected during routine care over a three-year period, and the reliability of any such analysis depends on how securely and consistently those records are captured and stored. The fact that our groups were separated in time is a reminder that documentation practices can drift, which is one of the harder biases to exclude in retrospective work. Digital tools for secure, traceable record-keeping are therefore of growing interest in orthopedics; blockchain-based systems, for example, have been proposed to preserve the integrity and provenance of clinical records, imaging, and registry data and to support multicenter data sharing [34]. A prospective, multicenter version of this study would be well placed to use such methods—time-stamped, tamper-evident capture of hemodynamic and pain data and standardized visualization scoring—to strengthen reproducibility and reduce the documentation-related bias inherent in a design like ours.

This study has several limitations. Most fundamentally, it was a retrospective, single-center, non-randomized cohort study whose control group came from a period before dexmedetomidine became routine, so allocation was defined by calendar period and the groups are separated in time. This introduces a risk of temporal bias—changes in surgical technique, anesthetic and perioperative care, learning-curve effects, and nursing protocols could all influence the outcomes and cannot be fully separated from the drug’s effect. We limited this by studying only isolated primary Bankart repairs performed by one experienced shoulder surgeon with an unchanged technique and fixed block and analgesia protocols across both periods, but residual confounding remains possible, and causal inference is not warranted. Because allocation was not randomized, selection bias cannot be excluded, and although patients who missed dexmedetomidine for a contraindication or supply shortage were excluded rather than moved to the control group, unmeasured differences between the eras may persist. The exclusion of the patients who received a titrated maintenance dose is itself a potential source of selection bias: it restricts the analysis to a specific, standardized fixed-dose regimen and means that the dexmedetomidine group is not representative of all patients who received the drug during the study period. Consequently, our findings apply only to the standardized fixed-dose regimen studied here (a 1 mcg/kg loading dose followed by 0.5 mcg/kg/h until the end of surgery). The analyses were also univariate: confounders not captured in routine records—operative duration, baseline anxiety, instability severity, number of prior dislocations, smoking, and preoperative pain—could not be adjusted for, so the associations may be partly confounded, and we have accordingly reported the findings as associations with confidence intervals rather than relying on significance alone. No a priori sample-size calculation was performed, as the cohort was fixed and retrospective; the study was therefore not designed to a pre-specified power target, and the confidence intervals should be used to judge the precision of each estimate. A single primary outcome (duration of analgesia) was pre-specified, and the principal outcomes survived Bonferroni correction, but most diastolic and mean arterial pressure differences did not and are reported as exploratory. Visualization was scored on a subjective, surgeon-rated scale from archived video rather than in real time; interobserver and intraobserver agreement on independent scores was high (κ = 0.807 and 0.778) but was not corroborated by objective measures such as bleeding volume. Finally, time to first rescue analgesia may reflect individual pain tolerance despite the standardized protocol. Balanced against these limitations, the study has clear strengths: a focused, homogeneous population of isolated primary Bankart repairs, a single surgeon and standardized technique, blinded visualization assessment, comprehensive hemodynamic reporting, and—to our knowledge—the first evaluation of adjunctive intravenous dexmedetomidine specifically in Bankart repair. The large, consistent effect sizes for the primary and key secondary outcomes are encouraging, but prospective, adequately powered randomized trials, ideally multicenter and with objective measures of bleeding, are needed to confirm them.

## 5. Conclusions

In conclusion, in this retrospective cohort of arthroscopic Bankart repairs, the addition of intravenous dexmedetomidine (1 mcg/kg loading dose followed by 0.5 mcg/kg/h infusion) to a single-shot interscalene block was associated with improved surgeon-rated visualization, lower postoperative pain scores, and prolonged analgesia compared with the block alone, together with lower intraoperative heart rate, lower diastolic blood pressure at 50 min mark and no increase in adverse events. Because of the retrospective, non-randomized design and the use of a historical control group, these results demonstrate association rather than causation and should not by themselves be taken to justify a change in routine practice. Prospective, adequately powered randomized controlled trials—ideally multicenter and incorporating objective measures of intraoperative bleeding—are required to confirm whether adjunctive intravenous dexmedetomidine causally improves surgical visualization and analgesia in arthroscopic shoulder surgery.

## Figures and Tables

**Table 1:** Demographic values and outcome values.

		Frequency (%)	Means (SD)	Min–Max	Median	*p*	Effect Size	r for Nonp.
Age	All Groups		25.33 (5.61)	15–43	25	0.965		
	Group D (33)		25.30 (6.32)	15–43	24			
	Group C (30)		25.37 (4.83)	16–36	25.5			
Gender								
Female		6 (9.5)						
Male		57 (90.5)						
Weight	All Groups		75.04 (11.10)	60–133	75	0.711		
	Group D (33)		74.54 (10.64)	60–102	75			
	Group C (30)		75.6 (11.74)	60–113	75.5			
ASA						0.211		
ASA 1		39 (61.9)						
ASA2		24 (38.1)						
Patient Satisfaction	All Groups		9.14 (0.8)	7–10	9	0.383 *		
	Group D (33)		9.24 (0.7)	7–10	9			
	Group C (30)		9.03 (0.88)	7–10	9			
Surgeon Satisfaction	All Groups		8.85 (1.18)	6–10	9	<*0.001* *		−0.518
	Group D (33)		9.42 (0.86)	6–10	10			
	Group C (30)		8.23 (1.19)	6–10	8			
VAS	All Groups		2.07 (1.64)	0–6	2	<*0.001*	1.882	
	Group D (33)		1 (1.08)	0–4	1			
	Group C (30)		3.26 (1.31)	1–6	3			
Duration of Analgesia (hours)	All Groups		5.88 (4.22)	1–24	5	*0.001*	0.848	
	Group D (33)		7.45 (4.56)	2–24	6			
	Group C (30)		4.16 (3.05)	1–12	3			
PCS	All Groups		12.36 (10.39)	0–42	8	0.810		
	Group D (33)		12.06 (10.82)	0–42	8			
	Group C (30)		12.7 (10.06)	0–34	8.5			
Heart Rate	All Groups		74.53 (11.27)	54–100	74	<*0.001*	1.08	
	Group D (33)		69.42 (11.55)	54–100	66			
	Group C (30)		80.16 (7.87)	67–97	80			

* Mann–Whitney U test was used for two independent groups; the rest of the *p*-values were calculated with an independent groups *t*-test. Statistically significant results are typed in italic font.

**Table 2:** Systolic blood pressure values.

	Patients (*n*)	Means (SD)	Min–Max	Median	*p*
SBP 0	All Groups	128.26 (12.51)	112–162	126	0.167
	Group D (33)	126.18 (12.13)	112–158	124	
	Group C (30)	130.57 (12.72)	112–162	130.50	
SBP 10	All Groups	121.73 (14.42)	94–169	123	0.513 *
	Group D (33)	121.06 (13.87)	94–169	120	
	Group C (30)	122.47 (15.20)	94–169	124.50	
SBP 20	All Groups	116.76 (11.04)	98–142	116	0.162 *
	Group D (33)	114.94 (10.27)	98–142	116	
	Group C (30)	118.77 (11.67)	99–142	119	
SBP 30	All Groups (62)	113.98 (10.93)	88–140	116	0.216
	Group D (33)	112.36 (10.99)	88–128	116	
	Group C (29)	115.83 (10.75)	95–140	117	
SBP 40	All Groups (59)	110.76 (12.71)	87–147	110	0.218
	Group D (33)	108.94 (10.81)	87–147	106	
	Group C (26)	113.08 (14.67)	87–147	110.5	
SBP 50	All Groups (46)	109.84 (9.36)	91–145	110	0.792
	Group D (23)	109.48 (6.25)	91–118	110	
	Group C (23)	110.22 (11.83)	91–145	110	
SBP 60	All Groups (8)	101.25 (4.36)	94–110	101	0.934
	Group D (6)	101.17 (5.15)	94–110	100.50	
	Group C (2)	101.50 (0.70)	101–102	101.50	

* Mann–Whitney U test was used for two independent groups; the rest of the *p*-values were calculated with an independent groups *t*-test.

**Table 3:** Diastolic blood pressure levels.

	Patients (*n*)	Means (SD)	Min–Max	Median	*p*	Effect Size
DBP 0	All Groups	71.98 (11.24)	57–98	72	0.108 *	
	Group D (33)	69.82 (10.97)	57–98	68		
	Group C (30)	74.37 (11.23)	57–90	76		
DBP 10	All Groups	64.63 (9.26)	50–86	63	0.707	
	Group D (33)	64.21 (8.24)	50–86	63		
	Group C (30)	65.10 (10.38)	50–84	63.50		
DBP 20	All Groups	60.56 (8.77)	41–82	61	*0.042*	0.521
	Group D (33)	58.42 (7.91)	41–80	58		
	Group C (30)	62.90 (9.21)	41–82	63.50		
DBP 30	All Groups (62)	60.02 (10.08)	40–80	58	*0.015*	0.637
	Group D (33)	57.12 (8.85)	40–80	58		
	Group C (29)	63.31 (10.51)	40–80	64		
DBP 40	All Groups (59)	57.86 (10.62)	38–84	58	*0.036*	0.561
	Group D (33)	55.30 (9.77)	38–84	56		
	Group C (26)	61.12 (10.94)	42–80	58		
DBP 50	All Groups (46)	57.07 (8.70)	40–77	55	*0.003*	0.936
	Group D (23)	53.35 (5.91)	43–66	51		
	Group C (23)	60.78 (9.54)	40–77	62		
DBP 60	All Groups (8)	54.38 (7.80)	42–62	57.50	0.523	
	Group D (6)	55.50 (6.83)	45–62	57.50		
	Group C (2)	51 (12.72)	42–60	51		

* Mann–Whitney U test was used for two independent groups; the rest of the *p*-values were calculated with an independent groups *t*-test. Statistically significant results are typed in italic font.

**Table 4:** MAP values.

	Patients (*n*)	Means (SD)	Min–Max	Median	*p*	Effect Size
MAP 0	All Groups	90.75 (10.33)	76–116	91	0.085	
	Group D (33)	88.61 (9.74)	76–116	87.67		
	Group C (30)	93.10 (10.60)	76–113	93.83		
MAP 10	All Groups	83.67 (9.29)	67–106	83	0.655	
	Group D (33)	83.16 (8.63)	67–106	83		
	Group C (30)	84.22 (10.08)	67–102	83		
MAP 20	All Groups	79.29 (8.15)	66–100	79.33	*0.037*	0.535
	Group D (33)	77.26 (7.29)	66–93	77.33		
	Group C (30)	81.52 (8.58)	66–100	82		
MAP 30	All Groups (62)	77.99 (9.59)	59–96	79	*0.032* *	−0.271
	Group D (33)	75.54 (8.72)	59–94	78		
	Group C (29)	80.79 (9.92)	59–96	80.33		
MAP 40	All Groups (59)	74.47 (12.62)	32–98	73	0.133	
	Group D (33)	72.27 (11.38)	32–95	72.33		
	Group C (26)	77.27 (13.77)	32–98	75.33		
MAP 50	All Groups (46)	74.66 (6.78)	60–89	73.33	*0.008*	0.822
	Group D (23)	72.06 (4.44)	66–81	70.33		
	Group C (23)	77.26 (7.76)	60–89	78		
MAP 60	All Groups (8)	70.04 (5.71)	61–75	73.67	0.857 *	
	Group D (6)	70.72 (5.40)	61–75	73.67		
	Group C (2)	68 (8.48)	62–74	68		

* Mann–Whitney U test was used for two independent groups; the rest of the *p*-values were calculated with an independent groups *t*-test. Statistically significant results are typed in italic font.

**Table 5:** Surgeon satisfaction regarding visual clarity.

Visual Clarity	Group D	Group C	*p*	Fisher Exact
Fair (*n* = 10)	1	9	0.003 *	0.005
Good (*n* = 53)	32	21		

* Chi-square test was used.

## Data Availability

The raw data supporting the conclusions of this article will be made available by the authors on request.

## Abbreviations

The following abbreviations are used in this manuscript:

ISBPB	Interscalene brachial plexus block
PCS	Pain Catastrophizing Scale
IV	Intravenous
SBP	Systolic blood pressure
DBP	Diastolic blood pressure
MAP	Mean Arterial Pressure
